# Sympathetic Neurons Regulate Cardiomyocyte Maturation in Culture

**DOI:** 10.3389/fcell.2022.850645

**Published:** 2022-03-11

**Authors:** William J. Kowalski, Iris H. Garcia-Pak, Wenling Li, Hideki Uosaki, Emmanouil Tampakakis, Jizhong Zou, Yongshun Lin, Kira Patterson, Chulan Kwon, Yoh-Suke Mukouyama

**Affiliations:** ^1^ Laboratory of Stem Cell and Neuro-Vascular Biology, Cell and Developmental Biology Center, National Heart, Lung, and Blood Institute, National Institutes of Health, Bethesda, MD, United States; ^2^ Division of Cardiology, Department of Medicine, Johns Hopkins University, Baltimore, MD, United States; ^3^ Division of Regenerative Medicine, Center for Molecular Medicine, Jichi Medical University, Shimotsuke, Japan; ^4^ IPSC Core Facility, National Heart, Lung, and Blood Institute, National Institutes of Health, Bethesda, MD, United States

**Keywords:** cardiomyocyte maturation, sympathetic innervation, iPS cells, heart development, co-culture

## Abstract

Embryos devoid of autonomic innervation suffer sudden cardiac death. However, whether autonomic neurons have a role in heart development is poorly understood. To investigate if sympathetic neurons impact cardiomyocyte maturation, we co-cultured phenotypically immature cardiomyocytes derived from human induced pluripotent stem cells with mouse sympathetic ganglion neurons. We found that 1) multiple cardiac structure and ion channel genes related to cardiomyocyte maturation were up-regulated when co-cultured with sympathetic neurons; 2) sarcomere organization and connexin-43 gap junctions increased; 3) calcium imaging showed greater transient amplitudes. However, sarcomere spacing, relaxation time, and level of sarcoplasmic reticulum calcium did not show matured phenotypes. We further found that addition of endothelial and epicardial support cells did not enhance maturation to a greater extent beyond sympathetic neurons, while administration of isoproterenol alone was insufficient to induce changes in gene expression. These results demonstrate that sympathetic neurons have a significant and complex role in regulating cardiomyocyte development.

## Introduction

The heart is densely innervated by sympathetic nerves, which extend from cell bodies housed in the sympathetic ganglia derived from trunk neural crest cells (Kimura et al., 2012). Sympathetic activity provides critical regulation of cardiac function, increasing beat rate, relaxation, contractile force, and conduction velocity (Hasan, 2013; Vegh et al., 2016). In cardiac pathologies, altered innervation density and sympathetic dysfunction are associated with heart failure, hypertension, arrhythmogenesis, and sudden cardiac death (Chen et al., 2007; Kimura et al., 2007; Florea and Cohn, 2014; Shen and Zipes, 2014; Fukuda et al., 2015). Furthermore, in pediatric patients, sympathetic defects have been linked to sudden infant death syndrome, certain congenital heart defects, cardiac arrhythmic death, and hypertension (Kahn et al., 2003; Crump et al., 2011; Ohuchi et al., 2011). These innervation disorders may have foundations in the interactions between cardiomyocytes (CMs) and sympathetic neurons (SNs) during development. Understanding the function of the cardiac sympathetic nervous system in the embryo and its role in heart development would give valuable insight into fetal, pediatric, and adult heart disease.

Neuronal projections first appear in the dorsal mesocardium at embryonic day (E) 10.5 in mice (Hildreth et al., 2009). At this point, embryonic CMs are already responsive to adrenergic stimulation (Vegh et al., 2016). Projections reach the aorta and pulmonary trunk at E11.5 and begin to differentiate into parasympathetic and sympathetic branches. By E13.5, sympathetic axons have started to innervate the ventricles, guided by nerve growth factor-expressing vascular smooth muscle of the coronary veins and later arteries (Nam et al., 2013). They continue to expand well into postnatal life, eventually rivaling the density of capillaries within the myocardium (Freeman et al., 2014; Zaglia and Mongillo, 2017). Disrupting cardiac sympathetic patterning in the embryo leads to postnatal arrhythmia and sudden death (Ieda et al., 2007), while abolishing sympathetic function results in prenatal death due to cardiac failure (Kobayashi et al., 1995; Thomas et al., 1995; Zhou et al., 1995; Rohrer et al., 1996; Baker et al., 2012). We have studied the autonomic devoid *Phox2b* ^*−/−*^ mouse and observed embryo mortality at E13.5-14.5 (Pattyn et al., 1999; Mokshagundam et al., 2021). We performed echocardiography to measure cardiac function prior to death, but found no progressive phenotype in *Phox2b* ^*−/−*^ embryos, suggesting that sudden death due to arrhythmia was the most likely cause. These studies demonstrate that the sympathetic system is essential for cardiac function and embryo survival. Despite their extensive innervation and essential role, the long-term effects of SNs on developing CMs are largely unknown, and it remains unclear if SNs influence aspects of CM maturation.

To investigate the role of SNs in CM maturation, we performed *in vitro* co-culture of human induced pluripotent stem cell (hiPSC)-derived CMs with embryonic mouse SNs. CMs derived from hiPSC display an immature, fetal-like phenotype and are a well-used model for studying CM maturation (Uosaki et al., 2015; Guo and Pu, 2020). We found that co-culture with SNs up-regulated multiple genes for CM maturation, ranging from myofiber components to ion channels and calcium machinery. Furthermore, gap junction expression increased, as well as sarcomere organization and intracellular Ca^2+^ transient amplitudes. Sarcomere spacing, however, was shorter and sarcoplasmic reticulum (SR) Ca^2+^ stores were reduced in co-cultured CM, demonstrating that SNs can also have a limiting effect on some aspects of maturation. We then incorporated endothelial cells (ECs) and epicardial-derived vascular smooth muscle and fibroblasts (EPI) into our co-culture model, as demonstrated in the recent studies that these cell types enhance maturation of hiPSC-derived CMs (Lee et al., 2015; Tan et al., 2021). We found that the maturation benefits of SNs were equal to or exceeded those of ECs and EPI, while combining all cell types did not produce further enrichment. Our results therefore indicate that SNs have a significant impact on developing CMs and play a role similar to other support cells in CM maturation.

## Materials and Methods

### Animals

C57BL/6J mice were purchased from the Jackson Laboratory. All experiments were performed under approval from the National Heart, Lung, and Blood Institute (NHLBI) Animal Care and Use Committee.

### hiPSC-CM Co-Culture

CMs were differentiated from ND2.0 hiPS cells expressing a GFP reporter as previously described (Chen et al., 2011; Luo et al., 2014; Lin et al., 2017). CMs were cryopreserved on day 10 and thawed 24 h before co-culture. SNs were obtained from dissected sympathetic ganglia of E13.5 mouse embryos as previously described (Nam et al., 2013). Sympathetic ganglia were dissociated to single cell suspension with the papain dissociation system according to the manufacturer’s directions (Worthington). Epicardial cells were harvested from E13.5 mouse embryo hearts as previously described (Nam et al., 2013). HUVECs were purchased commercially (Lonza). CMs and support cells were co-cultured at a ratio of 10:1. Culture surfaces were coated in 0.11 mg/ml growth factor reduced Matrigel (Corning). For FACS and qPCR, cells were cultured on 12 well plates (Falcon); for immunohistochemistry, cells were cultured on plastic coverslips (Nunc Thermanox); for Ca^2+^ imaging, cells were cultured on glass coverslips (Celltreat). Co-cultures were maintained in DMEM/F12 media (Gibco 11330) supplemented with 64 μg/ml L-ascorbic acid (Sigma),13.6 ng/ml selenium (Sigma), 10 μg/ml transferrin (Sigma), 5% chemically defined lipid concentrate (Gibco 11905), 5% fetal bovine serum (Hyclone), 1% penicillin-streptomycin (Gibco 15140), 20 μg/ml insulin (Sigma I9278), 20 ng/ml nerve growth factor (NGF, Millipore 01-125), 10 ng/ml human basic fibroblast growth factor (FGF-2, PromoCell C-60240), and 0.5 ng/ml human vascular endothelial growth factor-165 (VEGF_165_, PromoCell C-64420). Culture medium was changed every 2–3 days. All analyses were conducted on day 30 of cell culture.

### GCaMP6s Knock-in

GCaMP6s-expressing CMs were developed as follows. We used CRISPR/Cas9-mediated targeting to knock-in GCaMP6s at the AAVS1 safe harbor locus (Chen et al., 2013; Lin et al., 2018). The AAVS1 locus is an ideal target for the introduction of cell markers or other transgenes as it is actively and stably expressed in pluripotent stem cells as well as in differentiated cells (Patterson et al., 2020). Donor plasmid containing GCaMP6s and AAVS1 homology arms was transfected along with a AAVS1-CRISPR/Cas9 expression plasmid into 2 million ND2.0 iPSCs by nucleofection (Supplementary Figure S1A). Transfected iPSCs were seeded in E8 medium for 2 days, 0.5 μg/ml of puromycin (Sigma-Aldrich) was added on day 3 and continued for 10 days thereafter. The medium was changed every day. The survived colonies were picked and screened by PCR. Genomic DNA was collected from single colonies, and the gene targeting was confirmed by 5′-junction PCR (Supplementary Figure S1B). Primers AAVS1U-F2 (CTG​CCG​TCT​CTC​TCC​TGA​GT) and PuroU-R (GTG​GGC​TTG​TAC​TCG​GTC​AT) detected targeted allele. Primers AAVSEL-F (TTC​GGG​TCA​CCT​CTC​ACT​CC) and AAVSEL-R (GGC​TCC​ATC​GTA​AGC​AAA​CC) detected wild-type allele. A GCaMP6s heterozygous knock-in iPSC clone, which contains both knock-in allele and wild-type AAVS1 allele, was then differentiated into cardiomyocytes for evaluation of GFP fluorescent transients that reflects calcium flux during contraction (Supplementary Figure S1C). Normal karyotype of the gene-edited ND2.0-GCaMP6s iPSC clone was confirmed by G-band karyotyping (Supplementary Figure S1D).

### Flow Cytometry

Single-cell suspensions were prepared by treating cultured cells with 124.8 units/ml collagenase type 1 (Worthington LS004214), 147.4 units/ml collagenase type 2 (Worthington LS004202), 400 units/ml DNaseI (Sigma DN25), and 5 μM CaCl_2_ in DMEM/F-12 (Gibco 11330) for 30 min at 37°C with gentle pipetting. Following dissociation with collagenase, cells were treated with 0.05% Trypsin-EDTA (Gibco 25300) for 3 min at 37°C. Cells were then resuspended in Lebovitz’s L-15 medium containing 1 mg/ml bovine serum albumin (Sigma A9148), 10 mM HEPES (Gibco 15630), 1% penicillin-streptomycin (Gibco 15140), and 25 μg/ml DNaseI (Sigma DN25). Cells were passed through a 40 μm strainer (Falcon) prior to sorting. Cell viability was assessed with 7-aminoactinomycin D (Thermo Fisher Scientific, A1310). HUVECs were used as unstained and non-GFP controls. GFP^+^ hiPSC-CMs were isolated by FACS with the Aria II SORP instrument (BD Biosciences). CMs were sorted directly into TRIzol reagent (Invitrogen 15596026) and kept at −20°C until processing for RNA.

### qPCR Analysis

Total RNA was extracted from the cultured cells as previously described (Li et al., 2013). First-strand cDNAs were synthesized with SuperScriptIII reverse transcriptase (Invitrogen) using random hexamer primers according to the manufacturer’s instruction. The same starting mass of RNA was used for all experiments. Quantitative mRNA expression analysis was performed with LightCycler 96 (Roche) using FastStart Universal SYBR Green master (Roche). A 10 μl reaction was performed, with cDNA diluted 1:4 and a final primer concentration of 250 nM. Reactions were run in triplicates and a Ct difference greater than 0.5 cycles was rejected. At least *n* = 2 biological samples were analyzed per gene for each experimental group. Data was analyzed by the 2^−ΔΔCt^ method (Livak and Schmittgen, 2001). Glyceraldehyde 3-phosphate dehydrogenase (*GAPH*) and β-actin (*ACTB*) were used as housekeeper genes, with the geometric mean of their Ct values acting as the first normalization step (Vandesompele et al., 2002). These housekeeping genes were selected following stability analysis (Vandesompele et al., 2002). Data was then normalized to the CM-only control cultures as the second step. A detailed description of the PCR primers is described in Supplementary Table S1.

### Immunohistochemistry

Cell culture staining was performed as previously described (Nam et al., 2013). Cells were washed in cold PBS and fixed in 4% PFA/PBS for 15 min at room temperature. After washing in PBS, cells were incubated with primary antibodies overnight at 4°C in 0.1% Triton X-100/PBS with 10% heat inactivated goat serum. The following primary antibodies were used: bovine anti-cardiac troponin T antibody (mouse monoclonal, DSHB, 1:1,000) to detect CMs, rabbit anti-tyrosine hydroxylase antibody (Millipore, 1:200) to detect SNs, rabbit anti-Col1a1 antibody (Millipore, 1:500) to detect fibroblasts, Cy3-conjugated anti-αSMA antibody (mouse monoclonal antibody, clone1A4, Sigma, 1:1,000) to detect smooth muscle cells, rabbit anti-von Willebrand factor antibody (Sigma, 1:200) to detect endothelial cells, anti-α-actinin antibody (mouse monoclonal antibody, clone EA-53, Sigma, 1:500) to detect sarcomeres, and anti-Cx43 antibody (mouse monoclonal antibody, clone 2, BD Biosciences, 1:200) to detect gap junctions. For immunofluorescence detection, either Cy3-, Alexa-568-, or Alexa-647- conjugated secondary antibodies (Jackson ImmunoResearch or ThermoFisher Scientific, 1:250, 1 h at room temperature) were used. Coverslips were mounted with ProLong Gold Antifade Mounting solution (Thermo Fisher Scientific). All confocal microscopy was carried out on a Leica TCS SP5 confocal (Leica). To quantify sarcomere organization and spacing, sarcomeres were first segmented with ImageJ using a ridge detection algorithm (Steger, 1998). The segmented images were then partitioned into 20 × 20 μm windows. For each window, the local sarcomere organization and spacing were calculated using automated analysis in Matlab (Sutcliffe et al., 2018). Briefly, the co-occurrence matrix and Haralick correlation were computed for multiple angles, 0–180°, and offset distances, 0–3.8 μm, generating an m (number of angles) by n (number of offset distances) matrix of Haralick correlations (Haralick et al., 1973). The sarcomere score is the magnitude of the peak correlation, ranging between 0 and 2. The offset distance associated with the peak correlation is the sarcomere spacing. An idealized image of parallel stripes, all the same width, would give a maximum organization score of 2. After computing the organization and spacing for each window, the average values for the entire image was considered the overall sarcomere organization and spacing. Windows with no or insufficient sarcomeres were not included. Cx43 content was analyzed using custom Matlab scripts. Sample numbers are given in figure captions.

### Live Imaging and Quantification Analysis of GCaMP6s-hiPSC-CM

Intracellular Ca^2+^ transients were recorded from GCaMP6s-expressing CM-only and CM + SN cultured on glass coverslips. Cells were incubated at 37°C for 1 hour in fresh medium and then coverslips were transferred to an imaging chamber perfused at 1 ml/min with 37°C Tyrode’s solution containing (in mM) 145 NaCl, 5.4 KCl, 2 CaCl_2_, 0.5 MgCl_2_, 5.5 glucose, 5 HEPES, 0.3 NaH_2_PO4, pH adjusted to 7.4 (Knollmann et al., 2006). Propranolol (1 μM) was applied during pre-incubation and imaging to isolate CM from adrenergic signaling. Propranolol was used during CM-only imaging as well in order to maintain experimental parameters. Ca^2+^ imaging was performed with a Leica TCS SP5 confocal microscope (Leica). Time-lapse images were recorded at 22 frames/second with a 20X/0.7 NA objective. After several transients were recorded, 10 mM caffeine was applied by injecting a bolus directly into the imaging chamber. Caffeine was washed out for 5 min between image acquisitions. Ca^2+^ images were analyzed using custom Matlab scripts (Mathworks, Natick, MA). For each time series, three ROIs (7.5 × 7.5 μm) were manually selected for analysis. We analyzed *n* = 45 ROIs from 6 CM-only coverslips and *n* = 54 ROIs from 7 CM + SN coverslips.

### Statistics

Data are presented as mean ± standard deviation. Data were compared using upaired t-tests, Welch’s *t*-test, and Welch’s ANOVA with Dunnett’s T3 as indicated in the text and figure legends. *p* < 0.05 was considered significant. All statistical analysis was performed in GraphPad Prism (GraphPad Software, San Diego, CA).

## Results

### Cardiomyocytes, Sympathetic Neurons, and Other Support Cells Intermix in Co-Culture

**FIGURE 1:**
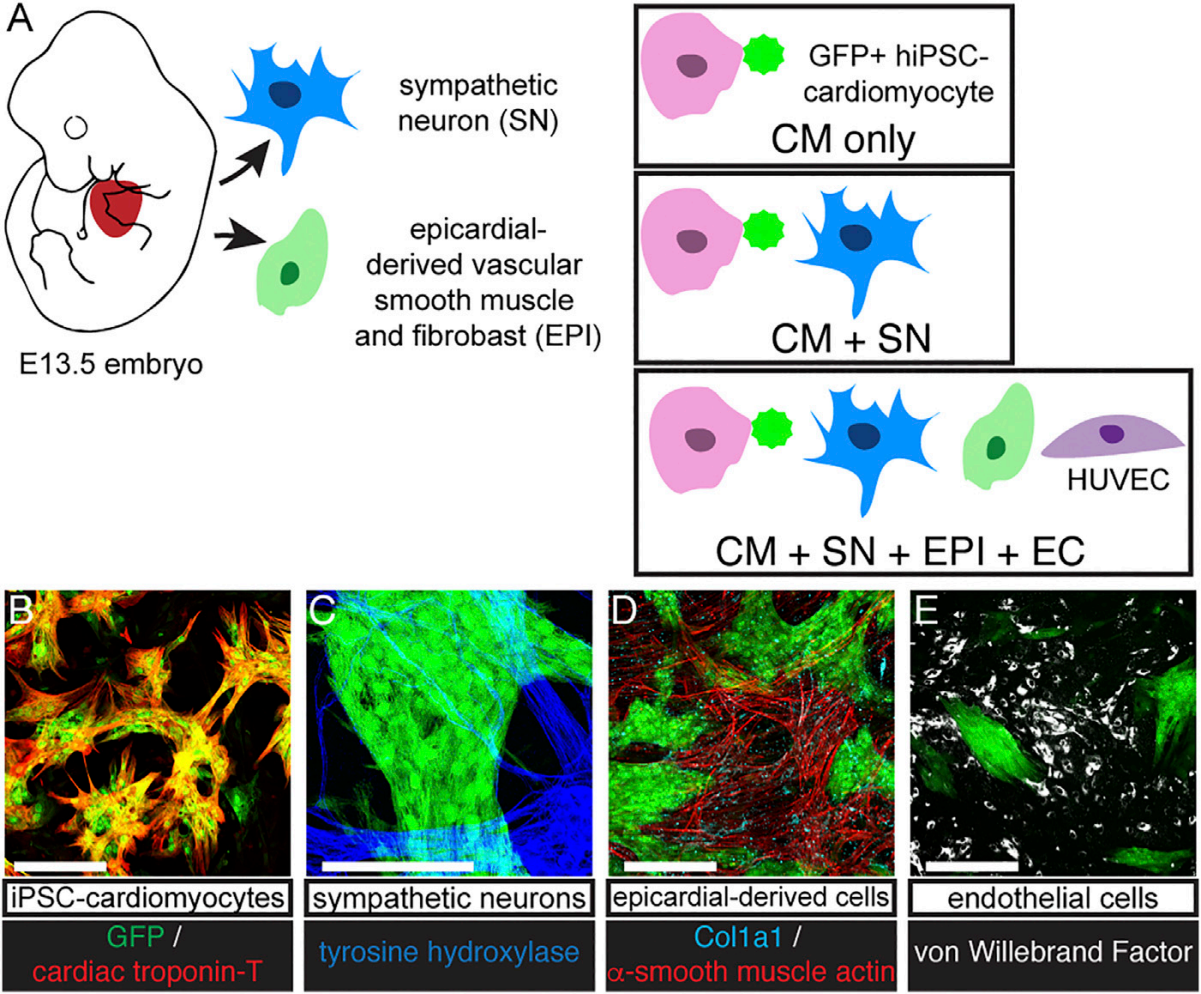
Co-culture protocol. **(A)** Diagram of cell sources and co-culture protocol. SNs were dissociated from E13.5 mouse sympathetic ganglia. GFP expressing hiPSC-CM were cultured alone (CM-only) or with SN (CM + SN) for 30 days. In other experiments, we included EPI and EC (CM + SN + EPI + EC). EPI were harvested from E13.5 mouse hearts and formed vascular smooth muscle and fibroblasts. ECs were HUVECS. **(B–E)** Co-cultures immunostained at day 30. **(B)** GFP-expressing hiSPC-derived CMs (green) were positive for cardiac troponin-T (red). **(C)** SNs expressed tyrosine hydroxylase (blue). **(D)** EPI cells showed α-smooth muscle actin (vascular smooth muscle, red) and Col1a1 (fibroblasts, cyan). **(E)** ECs are marked with von Willebrand factor (white). Scale bars 250 μm.

To understand how SNs support developing CMs, we established a long-term culture system (30 days) of GFP-expressing hiPSC-derived CMs (Lin et al., 2017) alone and with freshly isolated SNs dissected from E13.5 mouse embryos (Figure 1A). Cells were co-cultured on thin layers of Matrigel and grew to become 2–3 cell layers thick. CMs formed large clusters, which were surrounded by support cells (Figure 1). CMs expressed the GFP reporter and CM marker cardiac troponin T (cTnT, Figure 1B). After 30 days, we observed SNs with tyrosine hydroxylase immunostaining (Figure 1C). Multiple sympathetic axons and axon bundles extended toward and crossed into CM clusters, confirming that the two cell types were interwoven and not sequestered in our co-culture (Figure 1C, Supplementary Figure S2). We performed further experiments with EPI and ECs as additional support cells (Figure 1A). EPI is an epicardial-derived source of cardiac fibroblasts and coronary vascular smooth muscle cells. These support cells grew adjacent to the CM clusters, but we observed few non-CM within the clusters themselves (Figures 1D,E). EPI-derived cells expressed the fibroblast marker Col1a1 and vascular smooth muscle marker α-smooth muscle actin (α-SMA, Figure 1D). ECs expressed von Willebrand factor (Figure 1E). Throughout the 30 days co-cultures, CMs beat spontaneously.

### Sympathetic Neurons Up-Regulate Genes for Cardiomyocyte Maturation

**FIGURE 2:**
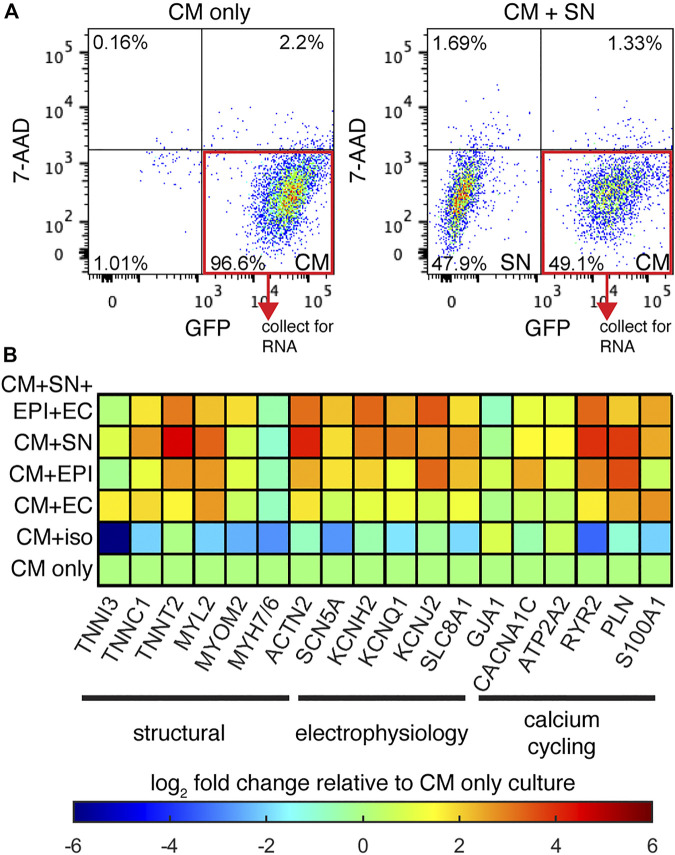
Relative gene expression. **(A)** Example FACS graphs of sorted GFP + CMs after dissociation of co-cultures. **(B)** Heatmap display of the log fold-change in CM specific genes, normalized to the CM-only culture. Genes are grouped based on their function in CM maturation. *n* ≥ 2 biological samples per gene.

We next examined changes in gene expression in co-cultured CM relative to CM-only controls. We performed quantitative PCR (qPCR) experiments on GFP^+^ CMs isolated from the co-cultures by FACS (Figure 2A). Relative expression is presented as a heatmap of log fold changes (Figure 2B). Genes tested were grouped into three categories: myofiber proteins, electrophysiology, and calcium cycling. When co-cultured with SNs, CMs showed increased expression of genes encoding structural components including *TNNC1*, *TNNT2*, *MYL2*, and *ACTN2*. Expression of ion channel genes such as *KCNH2*, *KCNQ1*, *KCNJ2*, *SCN5A*, and the Na^+^-Ca^2+^ exchanger (*SLC8A1*) were also increased, as well as genes encoding the calcium cycling machinery: *ATP2A2*, *RYR2*, *PLN*, *S100A1*. The gene encoding gap junction Cx43 (*GJA1*), however was not changed by co-culture with SNs. These data suggest that SNs can promote expression of genes for myofibril formation, electrophysiology functions, and calcium handling, all major facets of CM maturation.

We added additional support cells EPI and ECs to CM co-culture in order to better mimic the physiological environment. While CM + SN + EPI + EC showed up-regulation of multiple genes compared to CM-only controls, these additional support cells did not enhance expression beyond the changes observed in CM + SN (Figure 2B). In the case of some genes, such as *RYR2* and *PLN*, expression increased to greater levels when SNs were the only support cell. We then performed further co-cultures with a single support cell: CM + EPI and CM + EC. Co-culture with EPI as the single support cell increased gene expression as well, although the change for most genes was slightly less than the multi-support cell or CM + SN conditions. EC had little or no effect on CM gene expression. In our co-culture, therefore, additional support cells did not enhance the effects of SNs.

Our co-culture results indicated that SNs increased CM gene expression. We then examined whether SN-derived β-adrenergic signaling was the underlying factor, rather than the actual SN cells. To address this, we cultured CMs under chronic exposure to 1 μM isoproterenol, a non-selective β-agonist. Isoproterenol was added at each media change for the entire 30-days culture. Gene expression analysis revealed that the β-agonist reduced expression of several genes and had little to no effect on others. These results suggest that a different mechanism is responsible for the expression changes in CM + SN co-culture and that β-adrenergic signaling has no, or possibly a detrimental, effect on CM maturation.

### Sarcomere Maturation Is Slightly Delayed in Cardiomyocytes Co-Cultured With Sympathetic Neurons

**FIGURE 3:**
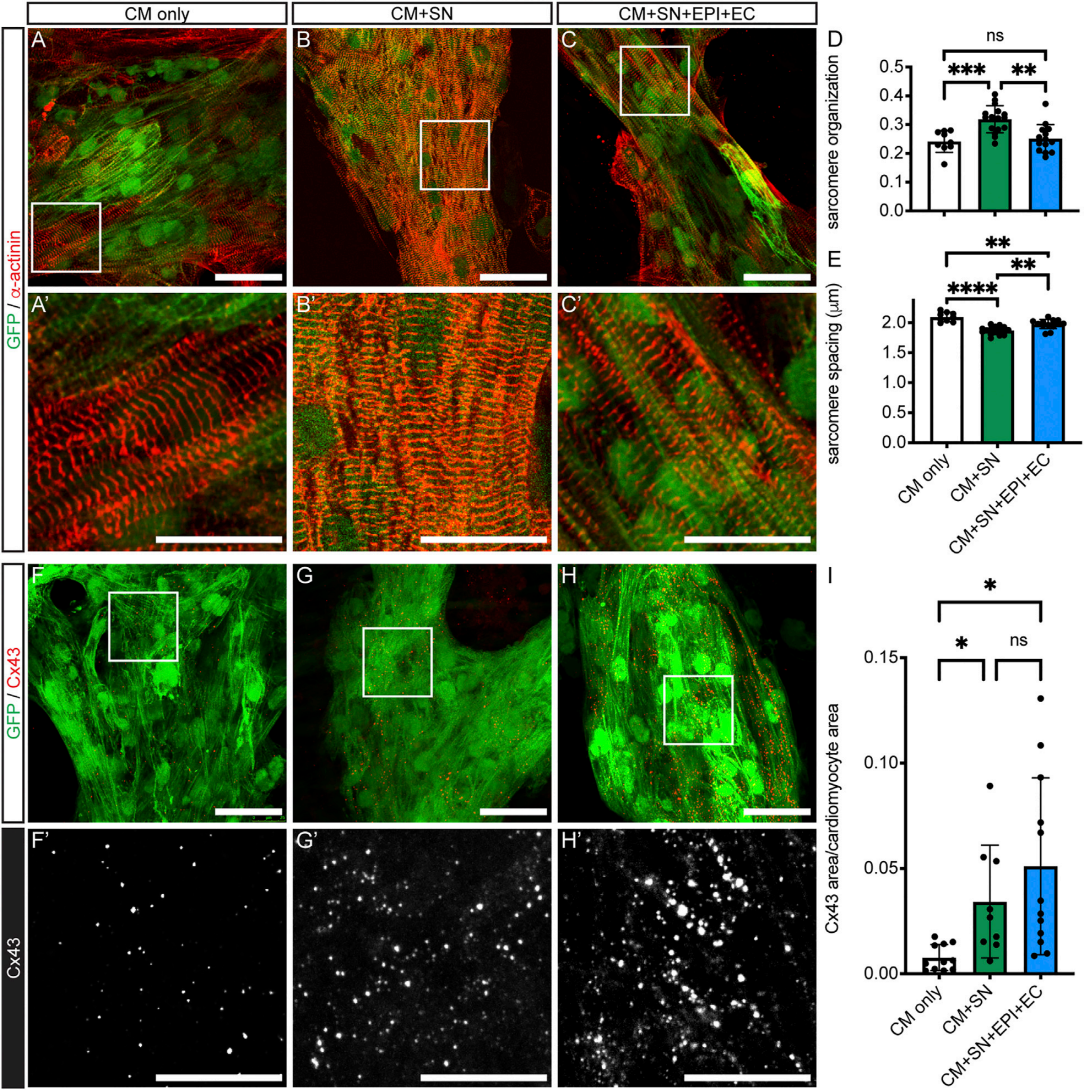
CM sarcomere organization and Cx43 expression. **(A–C)** Sarcomeres stained with α-actinin (red). GFP-expressing hiPSC-CM are shown in green. **(A′–C′)** Higher magnification of boxed areas in **(A–C)**. **(D–E)** Sarcomere organization **(D)** and spacing **(E)** from analysis of immunofluorescence images; **p <0.01, ***p <0.001, ****p <0.0001. Data are mean ± SD. Welch’s ANOVA with Dunnett’s T3 multiple comparisons test is shown. *n* = 9 areas from 3 CM-only cultures, *n* = 16 areas from 5 CM + SN, and *n* = 14 areas from 4 CM + SN + EPI + EC. **(F–H)** Immunofluorescence of Cx43 puncta (red) within hiPSC-CM (green). **(F′–H′)** Higher magnification of boxed regions in **(F–H)**. The Cx43 channel is shown in monochrome to visualize differences among the groups. **(I)** Total Cx43 area as a fraction of CM area; *p <0.05. Data are mean ± SD. Welch’s ANOVA with Dunnett’s T3 multiple comparisons test is shown. *n* ≥ 9 areas from five cultures per group. Scale bars 50 μm **(A–C)** and **(F–H)**, 25 μm **(A′–C′)** and **(F′–H′)**.

We next examined structural changes in the co-cultured CMs. We first analyzed sarcomere morphology in CMs with α-actinin staining (Figures 3A vs. 3B vs. 3C). CMs in all culture conditions showed parallel Z-disks distributed along the length of the cell. Sarcomere organization, which represents the alignment and regularity of spacing, was significantly increased in CM + SN (0.32 ± 0.05) compared to both CM-only controls (0.24 ± 0.04, *p* < 0.001) and CM + SN + EPI + EC (0.25 ± 0.05, *p* < 0.01, Welch’s ANOVA with Dunnett’s T3, Figure 3D). However, sarcomere spacing was significantly reduced by co-culture with SNs (CM + SN: 1.9 ± 0.07 μm, CM-only: 2.1 ± 0.08 μm, *p* < 0.0001, Figure 3E). When adding EPI and EC, sarcomere spacing recovered somewhat (2.0 ± 0.08 μm, *p* < 0.01, Figure 3E), but was still less than CM-only levels (*p* < 0.01). These additional cells may inhibit or counteract the effects of the SNs. In the developing heart, then, SNs may have a complex, dual role in sarcomere maturation, improving the alignment of Z-disks, but slightly delaying their spacing.

### Sympathetic Neurons Increase Cx43 in Cardiomyocytes

Gap junction formation is a critical step in CM maturation (Scuderi and Butcher, 2017). We performed the connexin 43 (Cx43) gap junction protein immunostaining and analyzed the amount of Cx43 relative to CM area (Figure 3I). Cx43 expression was significantly increased in CM + SN compared to CM-only controls (0.03 ± 0.03 CM + SN vs. 0.008 ± 0.006 CM-only, *p* < 0.05, Figure 3F vs. 3G,I). Cx43 levels did not increase further when EPI and EC were added along with SN (0.05 ± 0.04, *p* = 0.61, Figure 3F vs. 3H,I). Therefore, SNs may have an important role in promoting Cx43 formation in CMs. Interestingly, in the above-mentioned qPCR analysis, mRNA for the Cx43 gene (*GJA1*) did not show a large fold change relative to CM-only controls (Figure 2B). These results indicate that SNs might enhance translation or other post-transcriptional events, leading to greater levels of the Cx43 gap junction protein.

### Ca^2+^ Handling Is Changed Significantly by Co-Culture With Sympathetic Neurons

**FIGURE 4:**
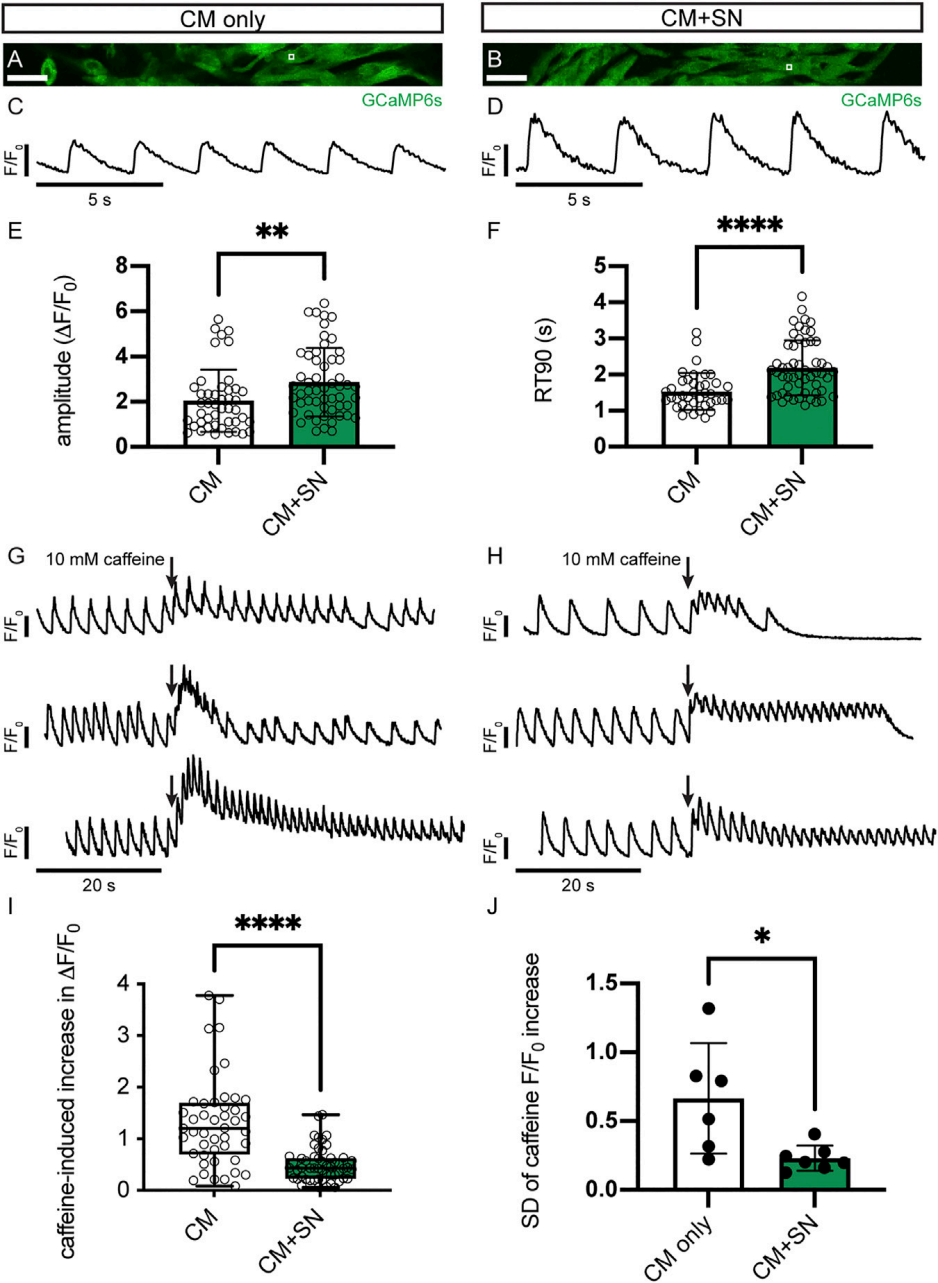
Ca^2+^ transients in CMs. **(A,B)** Sample images of GCaMP6s-expressing hiPSC-CMs in CM-only **(A)** and CM + SN **(B)** culture conditions. The white box regions show example ROIs for Ca^2+^ transient analysis. **(C)** Example trace of normalized Ca^2+^ fluorescence (F/F_0_) in CM-only culture during intrinsic beating. **(D)** Ca^2+^ trace of a CM + SN co-culture. **(E,F)** Amplitude **(E)** and RT90 **(F)** of the Ca^2+^ transients in CM-only and CM + SN; **p <0.01, ****p < 0.0001. Data are mean ± SD. Unpaired *t*-test is shown. **(G,H)** Example Ca^2+^ traces during caffeine injection in CM-only **(G)** and CM + SN **(H)** co-cultures. Three example plots are shown for both conditions to demonstrate the range of caffeine responses. Plots are scaled so that the baseline amplitudes appear equal. Arrows indicate the timing of caffeine infusion. **(I)** The caffeine-induced increase in Ca^2+^ transient amplitude was quantified relative to the baseline Ca^2+^ amplitude; ****p < 0.0001. Box plots extend between the 25th and 75th percentile, the median value is shown by a horizontal line, and the whiskers show the entire range. Welch’s *t*-test is shown. **(J)** Standard deviation (SD) of local caffeine increases observed across all ROIs from single coverslips; *p < 0.05. Data are mean ± SD. Unpaired *t*-test is shown. *n* = 45 ROIs from 6 CM-only and *n* = 54 ROIs from 7 CM + SN coverslips. Scale bars 100 μm.

To investigate functional changes in response to co-culture with SN, we analyzed intracellular Ca^2+^ transients during CM contraction (Figures 4A,B). GCaMP6s-expressing CMs and SNs were co-cultured on Matrigel-coated glass coverslips for 30 days and then imaged under perfusion with a warmed Tyrode’s buffer. Ca^2+^ imaging was conducted at the intrinsic, spontaneous beat rate of CMs (Figures 4C,D). We found that the amplitude of the Ca^2+^ transient was significantly greater in CMs co-cultured with SNs compared to CM-only controls (2.87 ± 1.52 CM + SN vs. 2.05 ± 1.38 CM-only, *p* < 0.01, unpaired *t*-test, Figure 4E). Time to 90% relaxation (RT90), however, was longer in CM + SN (2.20 ± 0.76 s vs. 1.54 ± 0.50 s, *p* < 0.0001, Figure 4F). The larger Ca^2+^ amplitude is indicative of a more mature phenotype, while longer RT90 suggests less mature Ca^2+^ clearance. Taken together, these data demonstrate that SNs have a significant, yet bi-directional, affect the development of CM Ca^2+^ transients.

We further examined development of the SR Ca^2+^ stores by administration of caffeine. In response to caffeine stimulus, both CM-only and CM + SN showed an increase in the Ca^2+^ peak followed by temporary cessation of Ca^2+^ flux (Figure 4G vs. 4H). The relative change in the caffeine peak compared to baseline was significantly reduced in CM + SN (0.50 ± 0.32 CM + SN vs. 1.32 ± 0.88 CM-only, *p* < 0.0001, Welch’s *t*-test, Figure 4G vs. 4H,I). This smaller caffeine response indicates that CMs co-cultured with SNs have significantly less mature SR Ca^2+^. The variability of the caffeine response, however, was significantly greater in CM-only controls (0.23 ± 0.09 CM + SN vs. 0.67 ± 0.40 CM-only, *p* < 0.05, unpaired *t*-test, Figure 4G vs. 4H,J). We measured this variability as the standard deviation across all Ca^2+^ ROIs in a single coverslip. These results show a significant role for SNs in development of the SR and its function in Ca^2+^ dynamics.

## Discussion

Multiple aspects of CM maturation were modified by co-culture with SNs, including gene expression, sarcomeres, gap junctions, and Ca^2+^ handling. SNs up-regulated genes important to the contractile apparatus, electrophysiology, and calcium machinery, improved sarcomere organization, generated greater Cx43 levels in cells, and increased Ca^2+^ transient amplitudes. These results suggest a positive effect of SNs on CM maturation. However, SNs were also responsible for shorter sarcomere lengths, longer Ca^2+^ relaxation, and decreased release of Ca^2+^ from the SR, indicating delayed maturation. This bimodal role may be important to regulate CM development *in vivo*. Incorporating EPI and ECs as additional support cells produced only modest changes, lending further support to a significant role for SNs in guiding CM maturation.

Relative gene expression by qPCR revealed that multiple genes related to CM maturation increased expression when co-cultured with SNs. Genes encoding both thin (*TNNC1*, *TNNT2*) and thick (*MYL2*) myofiber filaments were up-regulated in the presence of SNs. Additionally, *ACTN2*, which encodes the major Z-disk α-actinin protein, was increased. However, *TNNI3*, the mature isoform of troponin-I (Bedada et al., 2014), and the *MYH7*/*MYH6* ratio (Guo and Pu, 2020) showed only small effects, suggesting that these isoform switches may not be influenced by SNs. We furthermore found up-regulation of membrane bound ion channels, which are required to propagate the CM action potential. Genes for the major voltage-dependent Na^+^ channel (*SCN5A*) and inward rectifying K^+^ channel (*KCNJ2*) were both increased in CM + SN co-cultures. During the action potential, membrane depolarization triggers Ca^2+^-induced Ca^2+^ release, which facilitates contraction, followed by removal of Ca^2+^ from the cytoplasm as the CM relaxes (Bers, 2008). The main components for Ca^2+^ cycling all showed increased expression in CMs co-cultured with SNs, including L-type Ca^2+^ channel (*CACNA1C*), ryanodine receptor (*RYR2*), Na^+^-Ca^2+^ exchanger (*SLC8A1*), sarco/endoplasmic reticulum Ca^2+^-ATPase (SERCA, *ATP2A2*), and phospholamban (*PLN*). Interestingly, some of the largest expression increases occurred in *RYR2* and *PLN*, which may relate to the changes in Ca^2+^ dynamics in CM + SN discussed below. Taken together, these data show that SNs modify CM gene expression and promote a more mature profile.

We found that SNs increased sarcomere organization in CMs. Randomly oriented myofibrils with condensed Z-bodies is a hallmark of immature CMs (Snir et al., 2003; Kamakura et al., 2013; Lundy et al., 2013). In both CM-only and CM + SN, Z-disks are visible and mostly aligned to the CM long axis, similar to previous 30 days culture (Kamakura et al., 2013). In the presence of SNs, however, alignment has progressed further and sarcomeres are more uniformly spaced. These features can enhance the contractile force of CMs. Despite this increased organization, CMs co-cultured with SNs exhibited slightly shorter sarcomere lengths, indicating that SNs may inhibit sarcomere elongation. As CMs mature, sarcomere length–the distance between Z-disks–increases, reaching 2.2 μm in adult human CMs (Yang et al., 2014). We suggest, therefore, that SNs enhance organization but delay elongation of sarcomeres. Mechanisms of sarcomere assembly and maturation are slowly being uncovered (Guo et al., 2021), and further research is required to understand how these processes are regulated and interpret the effects of this dual role on CM function.

Cx43 increased in hiPSC-derived CMs co-cultured with SNs. Interestingly, the gene, *GJA1*, did not show increased expression, suggesting that SNs may regulate translation or another post-transcription mechanism. Cx43 forms the gap junctions in CMs, allowing for propagation of the action potential. In adult CMs, Cx43 is localized to the intercalated disc between individual cells. We did not observe any localization pattern in control or CM + SN. The effect of SN, therefore, seems limited to expression of the protein, but not trafficking to cell termini. In human CMs, Cx43 localization is a largely postnatal process and may take up to 7 years to completely organize at the intercalated disk (Vreeker et al., 2014). SN-promoted Cx43 expression, therefore, may be important to support later development of electrical conduction. A recent study using 3D hiPSC-derived CM/EC/fibroblast microtissues proposed that greater cAMP in CMs leads to increased Cx43 assembly (Giacomelli et al., 2020). Our study supports this role for sympathetic stimulation in CM development. That study further showed that decreased Cx43 expression was associated with arrhythmic behavior. If a lack of SNs leads to reduced Cx43 *in vivo*, this may give insight into the sudden cardiac embryolethality of *Phox2b* ^*−/−*^ and adrenergic-null mutants.

Our co-culture experiments showed that SNs significantly affect Ca^2+^ dynamics in hiPSC-derived CMs. We observed larger Ca^2+^ amplitudes in CM + SN vs. CM-only controls, but slower relaxation times. Previous studies have demonstrated that stem cell-derived CMs express Ca^2+^ handling proteins, although at a lower level than adult CMs (Satin et al., 2008). Our qPCR data showed increased expression of Ca^2+^ handling machinery such as *CACNA1C*, *SLC8A1*, *RYR2*, *ATP2A2*, and *PLN* in SN co-cultured hiPSC-CMs. While up-regulation of these genes is consistent with greater Ca^2+^ amplitudes, the reason for slower relaxation is less clear. A possible explanation is that the less mature SR Ca^2+^ stores in SN co-cultured CM may make these CMs more reliant on membrane bound Ca^2+^ channels for Ca^2+^ removal, prolonging their RT90. It is important to note, however, that we analyzed CMs at intrinsic beat rates, and the slower beat rates of CM + SN may contribute to the greater Ca^2+^ amplitudes (Figure 4). Field stimulation at different pacing rates would offer further insight into the effects of SN on Ca^2+^ cycling. Stem cell-derived CMs have been shown to exhibit functional features such as Ca^2+^-induced Ca^2+^ release and SERCA activity (Satin et al., 2008; Itzhaki et al., 2011; Hwang et al., 2015). In particular, it has been demonstrated *in vitro* that the caffeine triggered Ca^2+^ release increases with culture time as the CMs develop SR Ca^2+^ stores (Satin et al., 2008). However, in our study, we found that the caffeine Ca^2+^ release was significantly reduced in CM + SN. This caffeine response suggests that CM + SN have smaller SR Ca^2+^ stores compared to CM-only, indicating that SNs lead to a less mature SR Ca^2+^ phenotype. It is intriguing, however, that at baseline, CM + SN show a larger Ca^2+^ amplitude. If the CM + SN indeed have less SR Ca^2+^, this increase would likely be due to greater influx of Ca^2+^ through the L-type Ca^2+^ channel. An alternative scenario, though, is that CM + SN release a greater fraction of their SR Ca^2+^ during normal beating. More sensitive ryanodine channels is one possible mechanism. Upon introducing caffeine, there would be less reserve Ca^2+^, therefore resulting, in part, to the reduced caffeine amplitude. Additional assays testing the activation of ryanodine receptors and activity of the L-type Ca^2+^ channel could provide insight to this issue.

Although the magnitude of the caffeine triggered Ca^2+^ release was reduced, CM + SN showed a more consistent response to the caffeine stimulus. In CM-only cultures, the caffeine response varied greatly, with some CMs exhibiting only a modest caffeine peak while others showed a more than 3-fold change compared to their baseline amplitude. CM + SN, however, had a much narrower band of responses. This difference may reflect a role for SNs in regulating development of Ca^2+^ handling apparatuses. Without SNs, maturation of Ca^2+^ handling may progress more haphazardly, generating a heterogenous mix of CMs. With SNs, Ca^2+^ handling development may be more tightly controlled. An intriguing question is if more uniform Ca^2+^ handling and, possibly, lower SR Ca^2+^ could be protective against arrhythmia early in development. Without uniform Ca^2+^ handling, a variegated response to environmental stimuli, for example, could lead to discordant ventricular contraction or rhythmic defects. Additionally, delayed afterdepolarizations (DADs) occur when high luminal SR Ca^2+^ stimulates spontaneous Ca^2+^ release (Bers, 2008). These afterdepolarizations can trigger arrhythmias in some cases. With lower SR Ca^2+^, a DAD is less likely to occur. Later in development, these effects would need to dissipate, especially to grow the SR Ca^2+^. But there may be a window where SNs provide some protection to the developing heart. Further research can explore these potential roles of SNs.

The significance of non-CM cells in the heart has long been recognized and various cell types have been used to improve stem cell-derived CMs (Burridge et al., 2014; Lee et al., 2015; Masumoto et al., 2016; Scuderi and Butcher, 2017; Zhang et al., 2017; Giacomelli et al., 2020; Tan et al., 2021). To generate a more physiologic environment, we added EPI and EC to perform CM + SN + EPI + EC co-culture. CM + SN + EPI + EC showed improvements in gene expression and Cx43 level over CM-only controls. Compared to CM + SN, however, the addition of EPI and ECs did not have a strong effect. Changes in gene expression were similar, and in some cases genes were more highly expressed in CM + SN, such as *RYR2* and *PLN*. CM + EPI also achieved similar changes in gene expression compared to CM + SN + EPI + EC. In the case of Cx43, there was no difference when EPI and EC were added. We did note that the EPI and ECs may have countered the effects of the SNs on sarcomere organization and spacing, recovering both to at or near the CM-only levels. Together, these data suggest that multiple support cells do not add up to generate further maturation. Our CM + SN + EPI + EC results demonstrate that SNs can influence CM maturation similar to other, more widely studied, support cells.

SNs have received relatively little attention as a potential regulator of CM maturation. Most studies thus far have focused on their role in modulating CM proliferation, which remains unclear (Tampakakis and Mahmoud, 2021). Neonatal rat SN and CM co-culture improved contractility and increased L-type Ca^2+^ channel expression, which is in line with our findings (Lloyd and Marvin, 1990; Ogawa et al., 1992). Co-culture of stem cell-derived CMs with SNs have demonstrated that SNs can control CM beat rate, but effects on CM maturation were not investigated (Takeuchi et al., 2012; Sakai et al., 2017; Takayama et al., 2020). In summary, our study demonstrates that SNs have a significant impact on multiple facets of CM maturation, giving insight into their critical role during development.

Further research is required to understand how SNs act to influence CM maturation. Our qPCR results suggest that β-adrenergic signaling is not sufficient to achieve the changes we observe in CM + SN co-culture. A limited number of previous studies on β-adrenergic stimulation and immature CMs have left the issue mostly unsettled. Neonatal rat CMs showed decreased cell size but no effect on proliferation in response to isoproterenol treatment (Kreipke and Birren, 2015). In E11.5 mouse embryonic CMs, β-adrenergic stimulation inhibited proliferation *via* decreased phosphorylation of Erk and Akt (Feridooni et al., 2017). Studies with hiPSC-CMs have shown conflicting results with regards to β-adrenergic stimulation: it either results in CM hypertrophy (Zhi et al., 2012) or has no effect on cell size (Foldes et al., 2014). In the case of α-adrenergic signaling, the agonist phenylephrine promoted hypertrophy in hESC-CMs, but had no impact on hiSPC-CMs (Foldes et al., 2011; Foldes et al., 2014). In the clinical setting, chronic exposure to catecholamines contributes to progressive heart failure and be cardiotoxic (Lymperopoulos et al., 2013). A previous study exposing iPSC-CM engineered tissues to norepinephrine for 7 days showed features of heart failure, but these were significantly blunted in serum-containing media (Tiburcy et al., 2017). Our qPCR results showed reduced expression of several genes after 30-days exposure to isoproterenol. It is possible that these changes are due to heart failure development in hiPSC-CMs. However, few studies have examined the effects of chronic catecholamine exposure on immature CMs, and it will require additional experiments to determine if there are cardiotoxic effects.

The physical presence of SNs in our CM + SN co-cultures may be the significant factor modulating CM maturation, rather than adrenergic signaling. Multiple sympathetic axons extended toward and invaded CM clusters, potentially enabling cell-cell contacts. A previous study with neonatal rat CM and SN showed changes in CM morphology, synapsin puncta, and cadherin accumulation at sites of cell-cell overlap (Shcherbakova et al., 2007). If similar specialized zones occur in our co-culture model, it may allow for greater cell-cell interaction between SN and CM. Further experiments such as RNA-sequencing will be needed to understand what signaling pathways are changed in CM + SN vs. CM-only conditions. While a future such experiment may reveal new interactions between CM and SN, there remains much to be learned about adrenergic signaling. Although prior work has not shown a definitive role, it is clear from *Th* ^*−/-*^, *Dbh* ^*−/−*^, and other mouse models that catecholamines are essential for embryonic heart function and embryo survival (Thomas et al., 1995; Zhou et al., 1995; Baker et al., 2012; Osuala et al., 2012). In the *Phox2b* ^*−/−*^ model, embryos die mostly between E13.5-E14.5. At this stage, SNs have just reached the ventricle, innervating only a small portion of the dorsal subepicardium and are not yet in contact with CMs (Nam et al., 2013). Before SNs reach the heart, intrinsic cardiac adrenergic cells are required to sustain catecholamine levels (Ebert et al., 2008). In early embryonic CMs, therefore, adrenergic signaling is necessary, while the actual presence of SNs may be dispensable. Later CM development may require contact with SNs. SNs therefore likely act through a combination of adrenergic stimulation and local cell-cell signaling to influence CMs. As seen in the results of this study, SNs can have an enhancing or delaying effect on CM maturation.

In conclusion, we found that SNs had a significant influence on hiPSC-derived CM maturation. Gene expression, Cx43 quantity, sarcomere organization and some aspects of Ca^2+^ handling were improved in CM + SN co-culture, while maturation of sarcomere spacing and SR Ca^2+^ were delayed. Adding EPI and ECs as other support cells did not enhance maturation beyond CM + SN, highlighting the significance of SNs. We assessed that SNs act through more than cateholamines, as culture of CM with isoproterenol did not replicate the changes in gene expression. These data suggest a complex role for SN in CM development. Future research will examine the influence of SNs other facets of CM maturation. Understanding this relationship could provide insight into fetal and pediatric arrhythmias, in particular sudden cardiac death as well as other disorders.

## Data Availability

The original contributions presented in the study are included in the article/Supplementary Material, further inquiries can be directed to the corresponding author.

## References

[B1] Baker C. Taylor D. G. Osuala K. Natarajan A. Molnar P. J. Hickman J. (2012). Adrenergic Deficiency Leads to Impaired Electrical Conduction and Increased Arrhythmic Potential in the Embryonic Mouse Heart. Biochem. Biophysical Res. Commun. 423 (3), 536–541. 10.1016/j.bbrc.2012.05.163 22683331

[B2] Bedada F. B. Chan S. S.-K. Metzger S. K. Zhang L. Zhang J. Garry D. J. (2014). Acquisition of a Quantitative, Stoichiometrically Conserved Ratiometric Marker of Maturation Status in Stem Cell-Derived Cardiac Myocytes. Stem Cel Rep. 3 (4), 594–605. 10.1016/j.stemcr.2014.07.012 PMC4223713 25358788

[B3] Bers D. M. (2008). Calcium Cycling and Signaling in Cardiac Myocytes. Annu. Rev. Physiol. 70, 23–49. 10.1146/annurev.physiol.70.113006.100455 17988210

[B4] Burridge P. W. Metzler S. A. Nakayama K. H. Abilez O. J. Simmons C. S. Bruce M. A. (2014). Multi-cellular Interactions Sustain Long-Term Contractility of Human Pluripotent Stem Cell-Derived Cardiomyocytes. Am. J. Transl Res. 6 (6), 724–735. 25628783 PMC4297340

[B5] Chen G. Gulbranson D. R. Hou Z. Bolin J. M. Ruotti V. Probasco M. D. (2011). Chemically Defined Conditions for Human iPSC Derivation and Culture. Nat. Methods 8 (5), 424–429. 10.1038/nmeth.1593 21478862 PMC3084903

[B6] Chen L. S. Zhou S. Fishbein M. C. Chen P.-S. (2007). New Perspectives on the Role of Autonomic Nervous System in the Genesis of Arrhythmias. J. Cardiovasc. Electrophysiol. 18 (1), 123–127. 10.1111/j.1540-8167.2006.00590.x 16911576

[B7] Chen T.-W. Wardill T. J. Sun Y. Pulver S. R. Renninger S. L. Baohan A. (2013). Ultrasensitive Fluorescent Proteins for Imaging Neuronal Activity. Nature 499 (7458), 295–300. 10.1038/nature12354 23868258 PMC3777791

[B8] Crump C. Winkleby M. A. Sundquist K. Sundquist J. (2011). Risk of Hypertension Among Young Adults Who Were Born Preterm: a Swedish National Study of 636,000 Births. Am. J. Epidemiol. 173 (7), 797–803. 10.1093/aje/kwq440 21320866 PMC3105282

[B9] Ebert S. N. Rong Q. Boe S. Pfeifer K. (2008). Catecholamine-synthesizing Cells in the Embryonic Mouse Heart. Ann. N. Y Acad. Sci. 1148, 317–324. 10.1196/annals.1410.008 19120124 PMC4293025

[B10] Feridooni T. Hotchkiss A. Baguma-Nibasheka M. Zhang F. Allen B. Chinni S. (2017). Effects of β-adrenergic Receptor Drugs on Embryonic Ventricular Cell Proliferation and Differentiation and Their Impact on Donor Cell Transplantation. Am. J. Physiology-Heart Circulatory Physiol. 312 (5), H919–H931. 10.1152/ajpheart.00425.2016 PMC5451577 28283550

[B11] Florea V. G. Cohn J. N. (2014). The Autonomic Nervous System and Heart Failure. Circ. Res. 114 (11), 1815–1826. 10.1161/CIRCRESAHA.114.302589 24855204

[B12] Földes G. Matsa E. Kriston-Vizi J. Leja T. Amisten S. Kolker L. (2014). Aberrant α-Adrenergic Hypertrophic Response in Cardiomyocytes from Human Induced Pluripotent Cells. Stem Cel Rep. 3 (5), 905–914. 10.1016/j.stemcr.2014.09.002 PMC4235744 25418732

[B13] Földes G. Mioulane M. Wright J. S. Liu A. Q. Novak P. Merkely B. (2011). Modulation of Human Embryonic Stem Cell-Derived Cardiomyocyte Growth: a Testbed for Studying Human Cardiac Hypertrophy? J. Mol. Cell Cardiol. 50 (2), 367–376. 10.1016/j.yjmcc.2010.10.029 21047517 PMC3034871

[B14] Freeman K. Tao W. Sun H. Soonpaa M. H. Rubart M. (2014). *In Situ* three-dimensional Reconstruction of Mouse Heart Sympathetic Innervation by Two-Photon Excitation Fluorescence Imaging. J. Neurosci. Methods 221, 48–61. 10.1016/j.jneumeth.2013.09.005 24056230 PMC3858460

[B15] Fukuda K. Kanazawa H. Aizawa Y. Ardell J. L. Shivkumar K. (2015). Cardiac Innervation and Sudden Cardiac Death. Circ. Res. 116 (12), 2005–2019. 10.1161/CIRCRESAHA.116.304679 26044253 PMC4465108

[B16] Giacomelli E. Meraviglia V. Campostrini G. Cochrane A. Cao X. van Helden R. W. J. (2020). Human-iPSC-Derived Cardiac Stromal Cells Enhance Maturation in 3D Cardiac Microtissues and Reveal Non-cardiomyocyte Contributions to Heart Disease. Cell Stem Cell 26 (6), 862–879. e811. 10.1016/j.stem.2020.05.004 32459996 PMC7284308

[B17] Guo Y. Cao Y. Jardin B. D. Sethi I. Ma Q. Moghadaszadeh B. (2021). Sarcomeres Regulate Murine Cardiomyocyte Maturation through MRTF-SRF Signaling. Proc. Natl. Acad. Sci. USA 118 (2), e2008861118. 10.1073/pnas.2008861118 33361330 PMC7812832

[B18] Guo Y. Pu W. T. (2020). Cardiomyocyte Maturation. Circ. Res. 126 (8), 1086–1106. 10.1161/CIRCRESAHA.119.315862 32271675 PMC7199445

[B19] Haralick R. M. Shanmugam K. Dinstein I. H. (1973). Textural Features for Image Classification. IEEE Trans. Syst. Man. Cybern. SMC-3, 610–621. 10.1109/tsmc.1973.4309314

[B20] Hasan W. (2013). Autonomic Cardiac Innervation. Organogenesis 9 (3), 176–193. 10.4161/org.24892 23872607 PMC3896589

[B21] Hildreth V. Anderson R. H. Henderson D. J. (2009). Autonomic Innervation of the Developing Heart: Origins and Function. Clin. Anat. 22 (1), 36–46. 10.1002/ca.20695 18846544

[B22] Hwang H. S. Kryshtal D. O. Feaster T. K. Sánchez-Freire V. Zhang J. Kamp T. J. (2015). Comparable Calcium Handling of Human iPSC-Derived Cardiomyocytes Generated by Multiple Laboratories. J. Mol. Cell Cardiol. 85, 79–88. 10.1016/j.yjmcc.2015.05.003 25982839 PMC4530041

[B23] Ieda M. Kanazawa H. Kimura K. Hattori F. Ieda Y. Taniguchi M. (2007). Sema3a Maintains normal Heart Rhythm through Sympathetic Innervation Patterning. Nat. Med. 13 (5), 604–612. 10.1038/nm1570 17417650

[B24] Itzhaki I. Rapoport S. Huber I. Mizrahi I. Zwi-Dantsis L. Arbel G. (2011). Calcium Handling in Human Induced Pluripotent Stem Cell Derived Cardiomyocytes. PLoS One 6 (4), e18037. 10.1371/journal.pone.0018037 21483779 PMC3069979

[B25] Kahn A. Groswasser J. Franco P. Scaillet S. Sawaguchi T. Kelmanson I. (2003). Sudden Infant Deaths: Stress, Arousal and SIDS. Early Hum. Dev. 75 (Suppl. l), S147–S166. 10.1016/j.earlhumdev.2003.08.018 14693401

[B26] Kamakura T. Makiyama T. Sasaki K. Yoshida Y. Wuriyanghai Y. Chen J. (2013). Ultrastructural Maturation of Human-Induced Pluripotent Stem Cell-Derived Cardiomyocytes in a Long-Term Culture. Circ. J. 77 (5), 1307–1314. 10.1253/circj.cj-12-0987 23400258

[B27] Kimura K. Ieda M. Fukuda K. (2012). Development, Maturation, and Transdifferentiation of Cardiac Sympathetic Nerves. Circ. Res. 110 (2), 325–336. 10.1161/CIRCRESAHA.111.257253 22267838

[B28] Kimura K. Ieda M. Kanazawa H. Yagi T. Tsunoda M. Ninomiya S.-i. (2007). Cardiac Sympathetic Rejuvenation. Circ. Res. 100 (12), 1755–1764. 10.1161/01.RES.0000269828.62250.ab 17495227

[B29] Knollmann B. C. Chopra N. Hlaing T. Akin B. Yang T. Ettensohn K. (2006). Casq2 Deletion Causes Sarcoplasmic Reticulum Volume Increase, Premature Ca2+ Release, and Catecholaminergic Polymorphic Ventricular Tachycardia. J. Clin. Invest. 116 (9), 2510–2520. 10.1172/JCI29128 16932808 PMC1551934

[B30] Kobayashi K. Morita S. Sawada H. Mizuguchi T. Yamada K. Nagatsu I. (1995). Targeted Disruption of the Tyrosine Hydroxylase Locus Results in Severe Catecholamine Depletion and Perinatal Lethality in Mice. J. Biol. Chem. 270 (45), 27235–27243. 10.1074/jbc.270.45.27235 7592982

[B31] Kreipke R. E. Birren S. J. (2015). Innervating Sympathetic Neurons Regulate Heart Size and the Timing of Cardiomyocyte Cell Cycle Withdrawal. J. Physiol. 593 (23), 5057–5073. 10.1113/JP270917 26420487 PMC4667004

[B32] Lee D. S. Chen J.-H. Lundy D. J. Liu C.-H. Hwang S.-M. Pabon L. (2015). Defined MicroRNAs Induce Aspects of Maturation in Mouse and Human Embryonic-Stem-Cell-Derived Cardiomyocytes. Cel Rep. 12 (12), 1960–1967. 10.1016/j.celrep.2015.08.042 26365191

[B33] Li W. Kohara H. Uchida Y. James J. M. Soneji K. Cronshaw D. G. (2013). Peripheral Nerve-Derived CXCL12 and VEGF-A Regulate the Patterning of Arterial Vessel Branching in Developing Limb Skin. Developmental Cel 24 (4), 359–371. 10.1016/j.devcel.2013.01.009 PMC3591512 23395391

[B34] Lin Y. Linask K. L. Mallon B. Johnson K. Klein M. Beers J. (2017). Heparin Promotes Cardiac Differentiation of Human Pluripotent Stem Cells in Chemically Defined Albumin-free Medium, Enabling Consistent Manufacture of Cardiomyocytes. Stem Cell Transl Med 6 (2), 527–538. 10.5966/sctm.2015-0428 PMC5442822 28191759

[B35] Lin Y. Liu H. Klein M. Ostrominski J. Hong S. G. Yada R. C. (2018). Efficient Differentiation of Cardiomyocytes and Generation of Calcium-Sensor Reporter Lines from Nonhuman Primate iPSCs. Sci. Rep. 8 (1), 5907. 10.1038/s41598-018-24074-y 29651156 PMC5897327

[B36] Livak K. J. Schmittgen T. D. (2001). Analysis of Relative Gene Expression Data Using Real-Time Quantitative PCR and the 2−ΔΔCT Method. Methods 25 (4), 402–408. 10.1006/meth.2001.1262 11846609

[B37] Lloyd T. Marvin W. J. Jr. (1990). Sympathetic Innervation Improves the Contractile Performance of Neonatal Cardiac Ventricular Myocytes in Culture. J. Mol. Cell Cardiol. 22 (3), 333–342. 10.1016/0022-2828(90)91466-k 2355399

[B38] Lundy S. D. Zhu W.-Z. Regnier M. Laflamme M. A. (2013). Structural and Functional Maturation of Cardiomyocytes Derived from Human Pluripotent Stem Cells. Stem Cell Development 22 (14), 1991–2002. 10.1089/scd.2012.0490 PMC3699903 23461462

[B39] Luo Y. Liu C. Cerbini T. San H. Lin Y. Chen G. (2014). Stable Enhanced green Fluorescent Protein Expression after Differentiation and Transplantation of Reporter Human Induced Pluripotent Stem Cells Generated by AAVS1 Transcription Activator-like Effector Nucleases. Stem Cell Transl Med 3 (7), 821–835. 10.5966/sctm.2013-0212 PMC4073825 24833591

[B40] Lymperopoulos A. Rengo G. Koch W. J. (2013). Adrenergic Nervous System in Heart Failure. Circ. Res. 113 (6), 739–753. 10.1161/CIRCRESAHA.113.300308 23989716 PMC3843360

[B41] Masumoto H. Nakane T. Tinney J. P. Yuan F. Ye F. Kowalski W. J. (2016). The Myocardial Regenerative Potential of Three-Dimensional Engineered Cardiac Tissues Composed of Multiple Human iPS Cell-Derived Cardiovascular Cell Lineages. Sci. Rep. 6, 29933. 10.1038/srep29933 27435115 PMC4951692

[B42] Mokshagundam D. Kowalski W. Garcia-Pak I. Klaunberg B. Nam J. Mukouyama Y.-s. (2021). Ultrahigh-Frequency Echocardiography of Autonomic Devoid Phox2B Homozygous Embryos Does Not Reveal a Significant Cardiac Phenotype before Embryo Death. Ultrasound Med. Biol. 47 (3), 751–758. 10.1016/j.ultrasmedbio.2020.11.008 33293111 PMC8520219

[B43] Nam J. Onitsuka I. Hatch J. Uchida Y. Ray S. Huang S. (2013). Coronary Veins Determine the Pattern of Sympathetic Innervation in the Developing Heart. Development 140 (7), 1475–1485. 10.1242/dev.087601 23462468 PMC3596991

[B44] Ogawa S. Barnett J. V. Sen L. Galper J. B. Smith T. W. Marsh J. D. (1992). Direct Contact between Sympathetic Neurons and Rat Cardiac Myocytes *In Vitro* Increases Expression of Functional Calcium Channels. J. Clin. Invest. 89 (4), 1085–1093. 10.1172/JCI115688 1313444 PMC442964

[B45] Ohuchi H. Negishi J. Miyake A. Sakaguchi H. Miyazaki A. Yamada O. (2011). Long-term Prognostic Value of Cardiac Autonomic Nervous Activity in Postoperative Patients with Congenital Heart Disease. Int. J. Cardiol. 151 (3), 296–302. 10.1016/j.ijcard.2010.05.062 20580104

[B46] Osuala K. Baker C. N. Nguyen H.-L. Martinez C. Weinshenker D. Ebert S. N. (2012). Physiological and Genomic Consequences of Adrenergic Deficiency during Embryonic/fetal Development in Mice: Impact on Retinoic Acid Metabolism. Physiol. Genomics 44 (19), 934–947. 10.1152/physiolgenomics.00180.2011 22911456 PMC3472461

[B47] Patterson K. Linask K. L. Beers J. Zou J. (2020). Generation of Two tdTomato Reporter Induced Pluripotent Stem Cell Lines (NHLBIi003-A-1 and NHLBIi003-A-2) by AAVS1 Safe Harbor Gene-Editing. Stem Cel Res. 42, 101673. 10.1016/j.scr.2019.101673 PMC7046126 31869686

[B48] Pattyn A. Morin X. Cremer H. Goridis C. Brunet J.-F. (1999). The Homeobox Gene Phox2b Is Essential for the Development of Autonomic Neural Crest Derivatives. Nature 399 (6734), 366–370. 10.1038/20700 10360575

[B49] Rohrer D. K. Desai K. H. Jasper J. R. Stevens M. E. Regula D. P. Jr. Barsh G. S. (1996). Targeted Disruption of the Mouse Beta1-Adrenergic Receptor Gene: Developmental and Cardiovascular Effects. Proc. Natl. Acad. Sci. 93 (14), 7375–7380. 10.1073/pnas.93.14.7375 8693001 PMC38992

[B50] Sakai K. Shimba K. Ishizuka K. Yang Z. Oiwa K. Takeuchi A. (2017). Functional Innervation of Human Induced Pluripotent Stem Cell-Derived Cardiomyocytes by Co-culture with Sympathetic Neurons Developed Using a Microtunnel Technique. Biochem. Biophysical Res. Commun. 494 (1-2), 138–143. 10.1016/j.bbrc.2017.10.065 29042197

[B51] Satin J. Itzhaki I. Rapoport S. Schroder E. A. Izu L. Arbel G. (2008). Calcium Handling in Human Embryonic Stem Cell-Derived Cardiomyocytes. Stem Cells 26 (8), 1961–1972. 10.1634/stemcells.2007-0591 18483424

[B52] Scuderi G. J. Butcher J. (2017). Naturally Engineered Maturation of Cardiomyocytes. Front. Cel Dev. Biol. 5, 50. 10.3389/fcell.2017.00050 PMC5418234 28529939

[B53] Shcherbakova O. G. Hurt C. M. Xiang Y. Dell'Acqua M. L. Zhang Q. Tsien R. W. (2007). Organization of β-adrenoceptor Signaling Compartments by Sympathetic Innervation of Cardiac Myocytes. J. Cel Biol 176 (4), 521–533. 10.1083/jcb.200604167 PMC2063986 17296797

[B54] Shen M. J. Zipes D. P. (2014). Role of the Autonomic Nervous System in Modulating Cardiac Arrhythmias. Circ. Res. 114 (6), 1004–1021. 10.1161/CIRCRESAHA.113.302549 24625726

[B55] Snir M. Kehat I. Gepstein A. Coleman R. Itskovitz-Eldor J. Livne E. (2003). Assessment of the Ultrastructural and Proliferative Properties of Human Embryonic Stem Cell-Derived Cardiomyocytes. Am. J. Physiology-Heart Circulatory Physiol. 285 (6), H2355–H2363. 10.1152/ajpheart.00020.2003 14613910

[B56] Steger C. (1998). An Unbiased Detector of Curvilinear Structures. IEEE Trans. Pattern Anal. Machine Intell. 20 (2), 113–125. 10.1109/34.659930

[B57] Sutcliffe M. D. Tan P. M. Fernandez-Perez A. Nam Y.-J. Munshi N. V. Saucerman J. J. (2018). High Content Analysis Identifies Unique Morphological Features of Reprogrammed Cardiomyocytes. Sci. Rep. 8 (1), 1258. 10.1038/s41598-018-19539-z 29352247 PMC5775342

[B58] Takayama Y. Kushige H. Akagi Y. Suzuki Y. Kumagai Y. Kida Y. S. (2020). Selective Induction of Human Autonomic Neurons Enables Precise Control of Cardiomyocyte Beating. Sci. Rep. 10 (1), 9464. 10.1038/s41598-020-66303-3 32528170 PMC7289887

[B59] Takeuchi A. Shimba K. Mori M. Takayama Y. Moriguchi H. Kotani K. (2012). Sympathetic Neurons Modulate the Beat Rate of Pluripotent Cell-Derived Cardiomyocytes *In Vitro* . Integr. Biol. 4 (12), 1532–1539. 10.1039/c2ib20060k 23080484

[B60] Tampakakis E. Mahmoud A. I. (2021). The Role of Hormones and Neurons in Cardiomyocyte Maturation. Semin. Cel Developmental Biol. 118, 136–143. 10.1016/j.semcdb.2021.03.026 PMC8434956 33931308

[B61] Tan J. J. Guyette J. P. Miki K. Xiao L. Kaur G. Wu T. (2021). Human iPS-Derived Pre-epicardial Cells Direct Cardiomyocyte Aggregation Expansion and Organization *In Vitro* . Nat. Commun. 12 (1), 4997. 10.1038/s41467-021-24921-z 34404774 PMC8370973

[B62] Thomas S. A. Matsumoto A. M. Palmiter R. D. (1995). Noradrenaline Is Essential for Mouse Fetal Development. Nature 374 (6523), 643–646. 10.1038/374643a0 7715704

[B63] Tiburcy M. Hudson J. E. Balfanz P. Schlick S. Meyer T. Chang Liao M.-L. (2017). Defined Engineered Human Myocardium with Advanced Maturation for Applications in Heart Failure Modeling and Repair. Circulation 135 (19), 1832–1847. 10.1161/CIRCULATIONAHA.116.024145 28167635 PMC5501412

[B64] Uosaki H. Cahan P. Lee D. I. Wang S. Miyamoto M. Fernandez L. (2015). Transcriptional Landscape of Cardiomyocyte Maturation. Cel Rep. 13 (8), 1705–1716. 10.1016/j.celrep.2015.10.032 PMC4662925 26586429

[B65] Vandesompele J. De Preter K. Pattyn F. Poppe B. Van Roy N. De Paepe A. (2002). Accurate Normalization of Real-Time Quantitative RT-PCR Data by Geometric Averaging of Multiple Internal Control Genes. Genome Biol. 3 (7), research00341. 10.1186/gb-2002-3-7-research0034 PMC126239 12184808

[B66] Végh A. Duim S. Smits A. Poelmann R. Ten Harkel A. DeRuiter M. (2016). Part and Parcel of the Cardiac Autonomic Nerve System: Unravelling its Cellular Building Blocks during Development. Jcdd 3 (3), 28. 10.3390/jcdd3030028 PMC5715672 29367572

[B67] Vreeker A. van Stuijvenberg L. Hund T. J. Mohler P. J. Nikkels P. G. J. van Veen T. A. B. (2014). Assembly of the Cardiac Intercalated Disk during Pre- and Postnatal Development of the Human Heart. PLoS One 9 (4), e94722. 10.1371/journal.pone.0094722 24733085 PMC3986238

[B68] Yang X. Pabon L. Murry C. E. (2014). Engineering Adolescence. Circ. Res. 114 (3), 511–523. 10.1161/CIRCRESAHA.114.300558 24481842 PMC3955370

[B69] Zaglia T. Mongillo M. (2017). Cardiac Sympathetic Innervation, from a Different point of (Re)view. J. Physiol. 595 (12), 3919–3930. 10.1113/JP273120 28240352 PMC5471365

[B70] Zhang W. Kong C. W. Tong M. H. Chooi W. H. Huang N. Li R. A. (2017). Maturation of Human Embryonic Stem Cell-Derived Cardiomyocytes (hESC-CMs) in 3D Collagen Matrix: Effects of Niche Cell Supplementation and Mechanical Stimulation. Acta Biomater. 49, 204–217. 10.1016/j.actbio.2016.11.058 27890729

[B71] Zhi D. Irvin M. R. Gu C. C. Stoddard A. J. Lorier R. Matter A. (2012). Whole-exome Sequencing and an iPSC-Derived Cardiomyocyte Model Provides a Powerful Platform for Gene Discovery in Left Ventricular Hypertrophy. Front. Gene 3, 92. 10.3389/fgene.2012.00092 PMC3361011 22654895

[B72] Zhou Q.-Y. Quaife C. J. Palmiter R. D. (1995). Targeted Disruption of the Tyrosine Hydroxylase Gene Reveals that Catecholamines Are Required for Mouse Fetal Development. Nature 374 (6523), 640–643. 10.1038/374640a0 7715703

