# Supplementary material for: Sympathetic Neurons Regulate Cardiomyocyte Maturation in Culture
Source: Front Cell Dev Biol. 2022 Mar 11;10:850645. doi: 10.3389/fcell.2022.850645 (PMC8961983; doi:10.3389/fcell.2022.850645)
Supplement: Supplementary file 1 [file Table1.DOCX]

**Table S1. Gene-specific oligonucleotide primers for qRT-PCR**

| **Gene** | **Sense (5’ to 3’)** | **Antisense (3’ to 5’)** |
| --- | --- | --- |
| *TNNI3* | CCAACTACCGCGCTTATGC | TCGAGGCGGAGATCTTAGATTT |
| *TNNC1* | CAGCTGACAGAAGAGCAGAAAAAT | TCACCTTGCCCAGCTCCTT |
| *TNNT2* | ACAGAGCGGAAAAGTGGGAAG | TCGTTGATCCTGTTTCGGAGA |
| *MYL2* | ACAGGGATGGCTTCATTGAC | CCTCCTCCTTGGAAAACCTC |
| *MYOM2* | TGCTTTTGCAGAGAAGAATCGTG | CTGACGTACTTGGCCTGCTC |
| *MYH7* | TCGTGCCTGATGACAAACAGGAGT | ATACTCGGTCTCGGCAGTGACTTT |
| *MYH6* | GATAGAGAGACTCCTGCGGC | CCGTCTTCCCATTCTCGGTT |
| *ACTN2* | CTGCTGCTTTGGTGTCAGAG | TTCCTATGGGGTCATCCTTG |
| *SCN5A* | AGCTGGCTGATGTGATGGTC | CACTTGTGCCTTAGGTTGCC |
| *KCNH2* | CAACCTGGGCGACCAGATAG | GGTGTTGGGAGAGACGTTGC |
| *KCNQ1* | CGCGGAAGCCTTACGATGT | GAACAGTGAGGGCTTCCCAAT |
| *KCNJ2* | GTGCGAACCAACCGCTACA | CCAGCGAATGTCCACACAC |
| *SLC8A1* | TCATAGCTGATCGGTTCATGTCC | CAGTTGTCTTGGTGGTCTCTC |
| *GJA1* | GGTGACTGGAGCGCCTTAG | GCGCACATGAGAGATTGGGA |
| *CANCA1C* | TGATTCCAACGCCACCAATTC | GAGGAGTCCATAGGCGATTACT |
| *ATP2A2* | CATCAAGCACACTGATCCCGT | CCACTCCCATAGCTTTCCCAG |
| *RYR2* | CATCGAACACTCCTCTACGGA | GGACACGCTAACTAAGATGAGGT |
| *PLN* | ACCTCACTCGCTCAGCTATAA | CATCACGATGATACAGATCAGCA |
| *S100A1* | AGGAGCTGAAAGAGCTGCTG | AGGGATAAGTGGGGTGAGGT |
| *GAPDH* | ACAGCCTCAAGATCATCAGCAA | CCATCACGCCACAGTTTCC |
| *ACTB* | GGCACCCAGCACAATGAAG | CCGATCCACACGGAGTACTTG |
