# Supplementary material for: Sympathetic Neurons Regulate Cardiomyocyte Maturation in Culture
Source: Front Cell Dev Biol. 2022 Mar 11;10:850645. doi: 10.3389/fcell.2022.850645 (PMC8961983; doi:10.3389/fcell.2022.850645)
Supplement: Supplementary file 2 [file Image2.PDF]

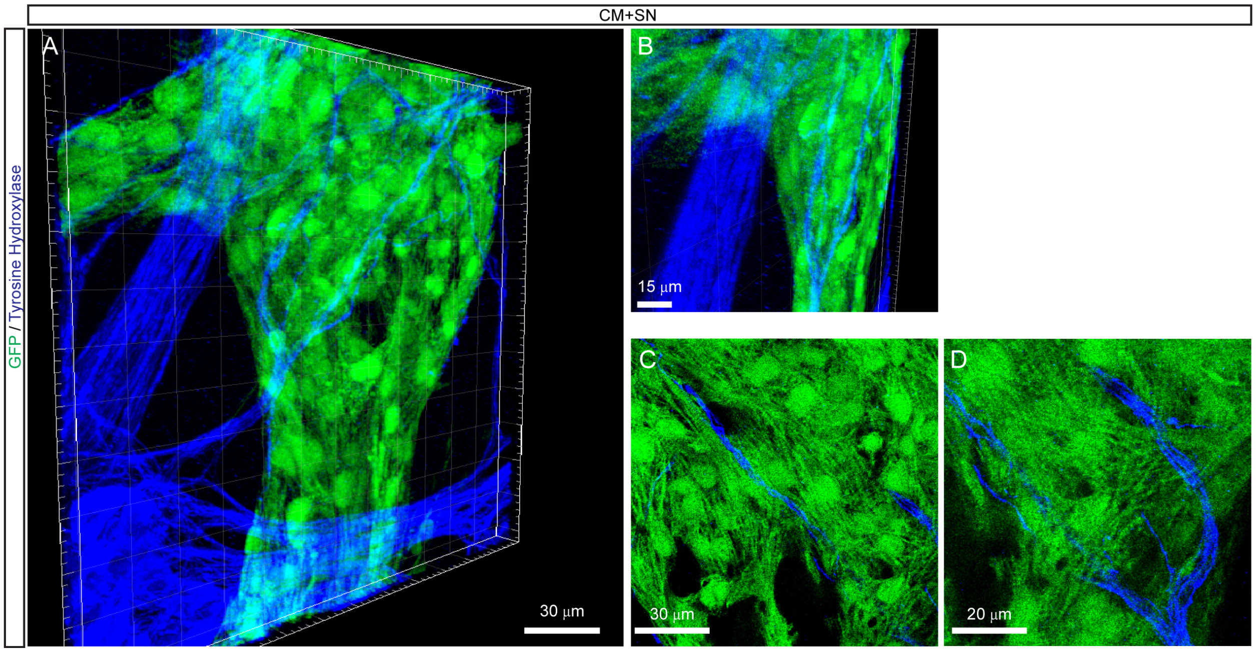

Figure S2. Detailed images of CM+SN co-culture in Figure 1C. Images show GFP+ CMs (green) and tyrosine hydroxylase expressing SN (blue). (A and B) 3D views show SNs extending through the middle and along the top and bottom of a CM cluster. (C and D) Higher magnification images show SN projections alongside CMs.
