# Supplementary material for: Sympathetic Neurons Regulate Cardiomyocyte Maturation in Culture
Source: Front Cell Dev Biol. 2022 Mar 11;10:850645. doi: 10.3389/fcell.2022.850645 (PMC8961983; doi:10.3389/fcell.2022.850645)
Supplement: Supplementary file 3 [file Image1.PDF]

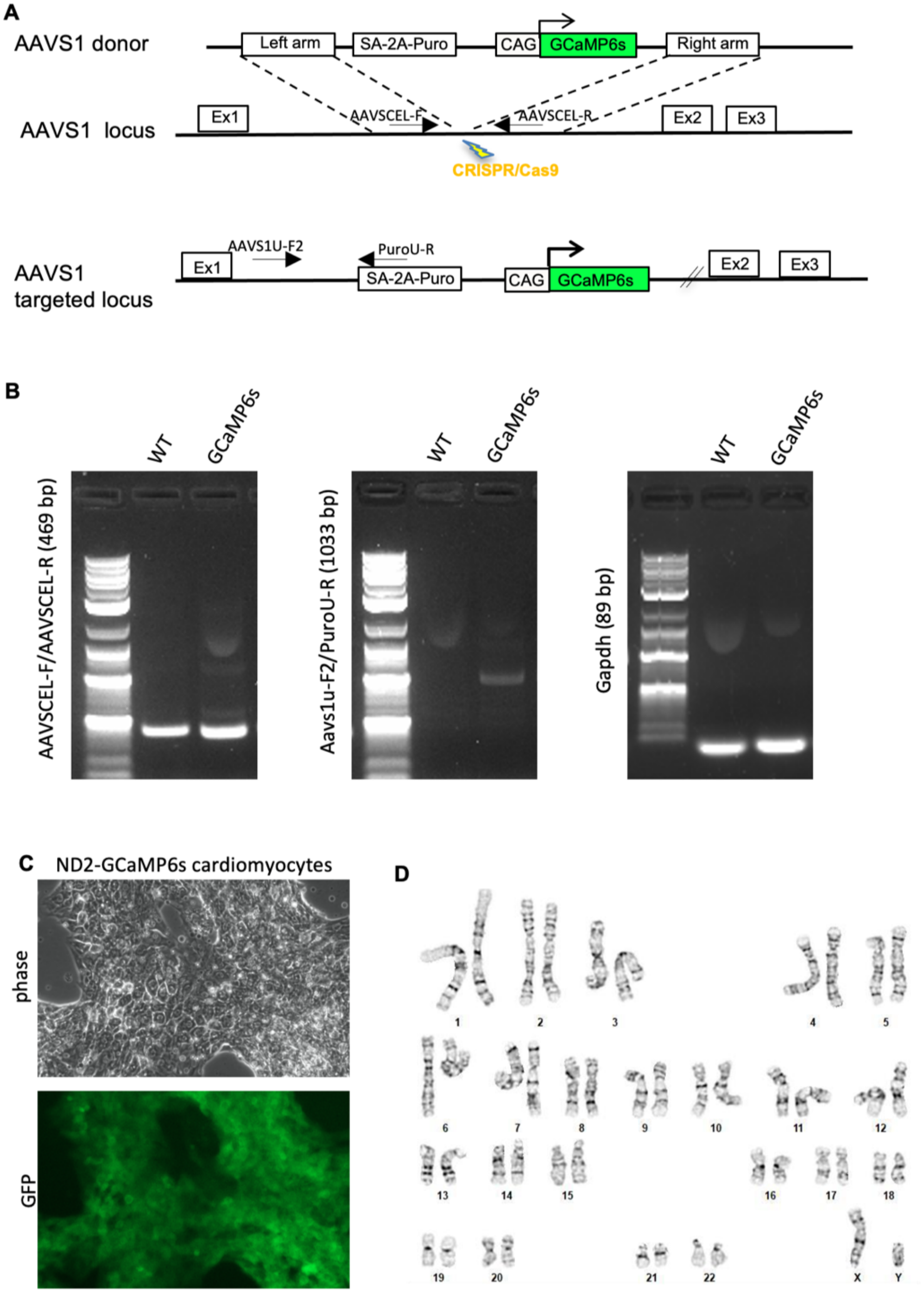

Figure S1. Genetically encoded calcium indicator (GECI) in ND2.0-iPSCs for detection of calcium flux in contracting cardiomyocytes. Related to Figure 4. (A) Schematic illustration of GCaMP6s knock-in at AAVS1 safe harbor mediated by CRISPR/Cas9 in human ND2.0 iPSCs. (B) 5'-junction and wild-type (WT) PCRs confirmed a heterozygous clone with knock-in of GCaMP6s at AAVS1 locus (C) Phase contrast and GFP-like fluorescence images of contracting cardiomyocytes derived from a ND2.0-GCaMP6s iPSC clone. (D) Normal karyotype of a ND2.0-GCaMP6s iPSC clone.
